# A ratiometric fluorescent sensor for Al^3+^ and Cu^2+^ detection in food samples

**DOI:** 10.3389/fnut.2025.1707179

**Published:** 2025-11-24

**Authors:** Mo Li, Xueguo Chen, Xinxin Xia, Ting Zhang, Wenji Zhang, Qingxin Xu, Xin Zhao

**Affiliations:** 1 College of Criminal Science and Technology, Criminal Investigation Police University of China, Shenyang, China; 2 School of Public Health, Shenyang Medical College, Shenyang, China

**Keywords:** fluorescence sensor, aluminum (III) ions, copper (II) ions, food samples, GODs

## Abstract

**Objective:**

Aluminum (Al) and copper (Cu), as two common elements, have specific applications in the modern food industry. However, excessive levels of Al^3+^ and Cu^2+^ pose a major risk to ecosystems and human health. Thus, fast, sensitive, and portable detection technologies are indispensable for achieving source control of such hazardous substances.

**Methods:**

We synthesized GSH-stabilized gold nanoclusters (G-AuNCs) and Nitrogen-doped graphene quantum dots (N-GQDs) via a straightforward hydrothermal method, and constructed GQDs@AuNCs ratiometric fluorescent sensors through electrostatic interactions for the rapid detection of Al^3+^ and Cu^2+^ in food samples.

**Results:**

When the concentration of Al^3+^ in the reaction medium increases, the fluorescence intensity (FI) of G-AuNCs incrementally enhances; in contrast, when the concentration of Cu^2+^ increases, the FI of G-AuNCs is gradually quenched. Notably, the FI of N-GQDs remains unchanged throughout this process. When the concentrations of Al^3+^ and Cu^2+^ ions are within the ranges of 0.5–500 μM and 1–200 μM, respectively, the FI ratio (I_528_/I_412_)/(I_528_/I_412_)_0_ shows a strong linear correlation with the ion concentrations, with corresponding limits of detection (LOD) of 0.66 μM and 0.44 μM.

**Conclusion:**

The fluorescent sensor features simple synthesis, rapid detection, and high sensitivity, making it well-suited for the rapid detection of Al^3+^ and Cu^2+^ in foods such as fried dough twists and shellfish.

## Introduction

In the modern food industry system, aluminum (Al) and copper (Cu) are two common elements with specific applications. Aluminum is widely used in food processing equipment and packaging materials due to its excellent thermal conductivity and ductility, while copper finds applications in food processing as a catalyst or in antimicrobial coatings owing to its biocidal properties (1, 2). Some food additives also contain aluminum elements, such as potassium aluminum sulfate (e.g., potassium alum) and ammonium aluminum sulfate (e.g., ammonium alum), which function as leavening agents, stabilizers, and pigment carriers in food production (3, 4). Copper is a trace element that is vital to human health, which is often added to specific foods as a nutritional fortifier (5). Unfortunately, excessive introduction of aluminum and copper ions into food can pose numerous potential risks to human health. Excessive aluminum intake has been closely linked to neurological damage, potentially leading to cognitive impairment, memory loss, and other issues, with more pronounced effects observed in elderly populations and children (6, 7). In the context of bone health, aluminum can disrupt calcium and phosphorus metabolism, and its long-term accumulation may give rise to disorders such as osteoporosis (8, 9). Additionally, it may impair hepatic and renal function, thereby affecting the body's metabolic and detoxification capabilities (10–12). Nowadays, various analytical methods have been used to detect Al^3+^ and Cu^2+^, including atomic absorption spectroscopy (AAS), Atomic fluorescence spectroscopy (AFS), and inductively coupled plasma optical emission spectrometer (ICP-OES). However, these methods are constrained by limitations including reliance on costly instruments, complicated sample pretreatment procedures, and restricted field applicability (13–15). Therefore, it is crucial to provide a practical and accurate analytical platform for the detection of Al^3+^ and Cu^2+^.

Currently, fluorescent sensors have garnered considerable research attention because of their special benefits, which include excellent sensitivity, ease of use, quick reaction, and real-time monitoring. Aydin et al. (16) constructed an aluminum ion (Al^3+^) responsive luminescent probe PHAB based on phenolphthalein, which enhances its emission by preventing the processes of C=N isomerization and photo-induced electron transfer (PET). In the course of this process, there is a noticeable hue shift from colorless to blue, making PHAB a fluorescent probe specific for Al^3+^ recognition. Wang et al. (17) synthesized a novel fluorescent probe PCTMT via the Suzuki reaction and Schiff base reaction, which exhibits excellent aggregation-induced emission (AIE) characteristics, high capacity to prevent interference with Co^2+^ and Cu^2+^ in aqueous media, together with excellent selectivity. Currently, most of the sensing systems that have been described for the detection of Al^3+^ and Cu^2+^ rely either on synthesized fluorescent probes or single-wavelength sensing systems. However, sensors with a single emission wavelength are susceptible to interferences from environmental factors (18). By contrast, dual-emission wavelength ratiometric fluorescent sensors can address these issues by measuring the intensity ratio of two emission peaks (19). Such sensors offer advantages including significant fluorescence intensity changes and naked-eye-visible color transitions, thereby enabling semi-quantitative detection of analytes. Thus, ratio fluorescence sensing represents a pivotal research frontier for next-generation fluorescent sensors.

Graphene quantum dots (GQDs) are nanomaterials with zero-dimensional longitudinal widths that are usually below 100 nm (20). GQDs with stable physical and chemical properties are one of the emerging carbon-based nanomaterials studied in recent years (21). GQDs exhibit low toxicity, good biocompatibility, and easy functionalization. Additionally, their fast electron transfer, excellent conductivity, and remarkable optical properties make GQDs ideal for optimizing electrocatalytic charge-transfer processes, facilitating effective electron transfer, and raising sensor ratios of signal to noise (22). These features endow CQDs with broad application prospects in identifying metal ions (23).

Recently, frequent incidents of excessive aluminum and copper in food have raised widespread public concern. Such risks exist in both street food and industrially processed products. Thus, accurate quantification of Al^3+^ and Cu^2+^ in food, in-depth investigation of contamination causes, and implementation of strict regulatory measures are critical for ensuring public dietary safety and protecting consumer health rights. In this study, N-GQDs and G-AuNCs were synthesized via a simple hydrothermal method. Ratiometric fluorescence sensors (GQDs@AuNCs) were assembled through electrostatic interactions for the detection of Cu^2+^ and Al^3+^ (The synthesis process and fluorescence color change are shown in Supplementary Figure S1). Visual detection of Cu^2+^ was achieved via fluorescence quenching by charge transfer mechanism (color change: initial purple → blue), while Al^3+^ detection relied on aggregation-induced emission by AIE (color change: initial purple → pink). The sensor features high sensitivity, rapid detection, and simple synthesis, enabling its application in the rapid quantification of Al^3+^ in Fried Dough Twists and deep-fried dough sticks and Cu^2+^ in scallops and *Sinonovacula constricta*.

## 2 Material and methods

### 2.1 Materials and reagents

Fried Dough Twists, deep-fried dough sticks, scallops and *Sinonovacula constricta* were acquired from a local shop in Shenyang, Liaoning, China. Graphene oxide (GO), Chloroauric acid trihydrate (HAuCl_4_·3H_2_O), Reduced Glutathione (GSH), Aqueous ammonia (NH_3_·H_2_O), Disodium Hydrogen Phosphate (NaH_2_PO_4_·2H_2_O), were acquired from Aladdin Reagent (Shanghai, China) Co., Ltd. Every other chemical was of the reagent kind.

### 2.2 Fabrication of N-GQDs

The fabrication of N-GQDs follows the method of Gao et al. and is slightly adjusted (24). Add 1g of GO powder to 30 mL of ultra-pure water, then add 20 mL of ammonia water (30%, v/v), stir thoroughly, and add the mixture to a high-pressure vessel lined with polytetrafluoroethylene. React at 160 °C for 8 h, cool the reaction solution to 25°C and filter. After refluxing the filtered liquid in a boiling state for 2 h, N-GQDs can be obtained.

### 2.3 Synthesis of G-AuNCs

The preparation of G-AuNCs follows the method of Wang et al. with slight modifications (25). First, mix 5 mL of GSH solution (10 mM) and 5 mL of HAuCl_4_ solution (10 mM) thoroughly. Then, stir the mixture for 24 h at 80 °C after adding 20 mL of ultrapure water. Once the solution has cooled to ambient temperature, centrifuge it for 5 min at 9,000 rpm. Finally, collect the supernatant and filter it to acquireG-AuNCs across a 0.22 μm membrane.

### 2.4 Synthesis of GQDs@AuNCs Nanocomposite

The GQDs@AuNCs nanocomposite was synthesized by assembling positively charged N-GQDs and negatively charged G-AuNCs through electrostatic interactions. Specifically, 5 mL of 5-fold diluted N-GQDs solution was thoroughly mixed with 3 mL of G-AuNCs solution to form the GQDs@AuNCs nanocomposite, thereby obtaining the GQDs@AuNCs ratiometric fluorescent sensor.

### 2.5 Characterization of GQDs@AuNCs

The microstructure of N-GQDs and G-AuNCs was characterized using a TEM. The N-GQDs and G-AuNCs solutions were thoroughly ultrasonically dispersed in an ultrasonic cleaner. After being spread out, the test solution was put onto a copper grid coated with carbon and allowed to air dry. The dried copper grid was placed in the sample chamber for morphological observation of N-GQDs and G-AuNCs.

The particle size distributions and zeta-potentials of N-GQDs and G-AuNCs were analyzed with a Zetasizer Nano-ZS90.

X-ray photoelectron spectroscopy was used to determine the elemental composition of GQDs@AuNCs. For sample preparation, a 5 mL centrifuge tube was filled with 2 mL of the GQDs@AuNCs nanocomposite and dried using a vacuum freeze dryer. Remove the processed GQDs@AuNCs nanocomposite and secure it onto the sample stage using double-sided conductive tape. Then, acquire the XPS spectrum of the GQDs@AuNCs nanocomposite.

The GQDs@AuNCs nanocomposite was analyzed by a FT-IR. The GQDs@AuNCs solution was drop-cast onto potassium bromide (KBr) powder. After being dried and ground, the mixture was pressed into a pellet. The pellet was then loaded into the FT-IR sample chamber for spectral acquisition to obtain the infrared spectrum of GQDs@AuNCs.

Take 100 μL of N-GQDs, 100 μL of G-AuNCs, and 100 μL of GQDs@AuNCs nanocomposite solution into centrifuge tubes, respectively. Add PBS buffer (pH = 7) to adjust the total volume to 1.5 mL, transfer each mixture to a quartz cuvette, place the cuvettes into the sample compartment of a fluorescence spectrophotometer for detection.

### 2.6 Sensitivity and selectivity assays of GQDs@AuNCs for Al^3+^ and Cu^2+^ detection

Transfer 200 μL of the GQDs@AuNCs nanocomposite solution and varying concentrations of Al^3+^ or Cu^2+^ into separate 5 mL centrifuge tubes. After adding PBS solution to adjust the total volume to 2 mL, determine the FI of the entire system. Next, create a calibration curve using the FI ratio (I_528_/I_412_)/(I_528_/I_412_)_0_ [where I_528_ and I_412_ represent the FI of G-AuNCs and N-GQDs, respectively, and (I_528_/I_412_) and (I_528_/I_412_)_0_ represent the FI ratios pre- and post-addition of the analyte], as well as the concentration of Al^3+^ or Cu^2+^.

The selectivity of the sensor was detected by adding different metal cations (Al^3+^, Cu^2+^, Fe^3+^, K^+^, Na^+^, Ca^2+^, Mn^2+^, Zn^2+^, Ba^2+^, Mg^2+^) to GQDs@AuNCs nanocomposites.

### 2.7 Detection of Al^3+^ and Cu^2+^ in practical samples

The prepared GQDs@AuNCs ratio fluorescent sensor was evaluated by using the standard addition recovery method to detect Al^3+^in deep-fried dough sticks and Fried Dough Twists, and Cu^2+^in scallop and *Sinonovacula constricta*. Pretreat the samples according to the method of Sun et al. (26), take 0.5 g of sample (deep-fried dough sticks, Fried Dough Twists, scallop, Sinovacula constricta) and add it to 5 mL concentrated nitric acid and 1 mL 30% hydrogen peroxide, mix thoroughly, and treat in a microwave for 20 min. Dilute to a constant volume of 50 mL with distilled water, add Al^3+^ and Cu^2+^ standard solutions (10 μM, 50 μM, 100 μM) as spiking solutions for the actual samples. The filtrate can be obtained by passing the prepared solution via a 0.22 μm filter membrane. Add the resulting filtrate to the GQDs@AuNCs nanocomposite, mix thoroughly, and measure using a fluorescence spectrophotometer.

## 3 Results and discussion

### 3.1 Structural characterization

**Figure 1:**
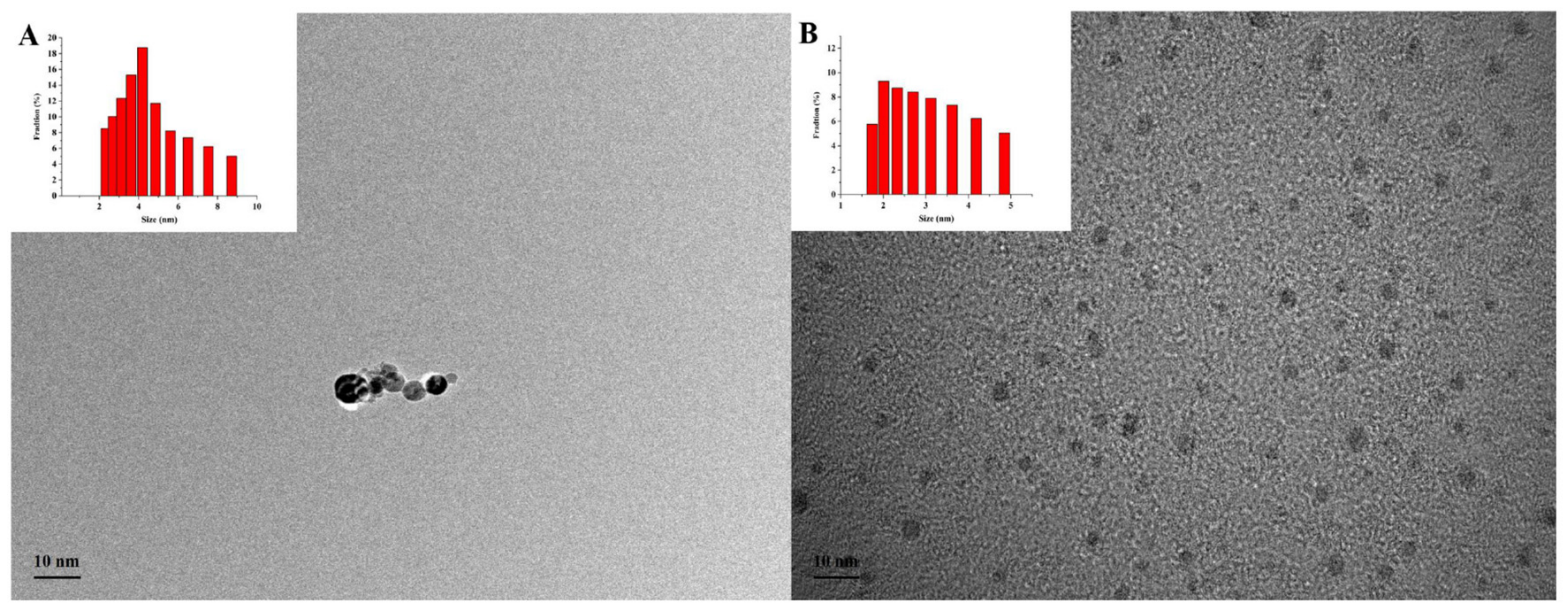
The microstructure of N-GQDs **(A)** and G-AuNCs **(B)**.

The synthesized N-GQDs andG-AuNCs were analyzed using a TEM and a laser force size analyzer. As shown in Figure 1, N-GQDs and G-AuNCs are spherical in shape and have good dispersibility. The average particle sizes of N-GQDs and G-AuNCs are 4.54 nm and 2.98 nm, respectively. The Zeta potentials of N-GQDs,G-AuNCs, and GQDs@AuNCs are 5.63 ± 0.65, −29.37 ± 0.77, and −7.92 ± 0.20 mV, respectively (Supplementary Figure S2). The positive potential of N-GQDs is attributed to the amino groups on their surface, while the negative charge ofG-AuNCs is attributed to the carboxyl groups in GSH (25). The charge of GQDs@AuNCs results from the interaction between the positive charge of N-GQDs and the negative charge of G-AuNCs, which also confirms that N-GQDs and G-AuNCs self-assemble through electrostatic interactions to form the GQDs@AuNCs nanocomposite (27).

**Figure 2:**
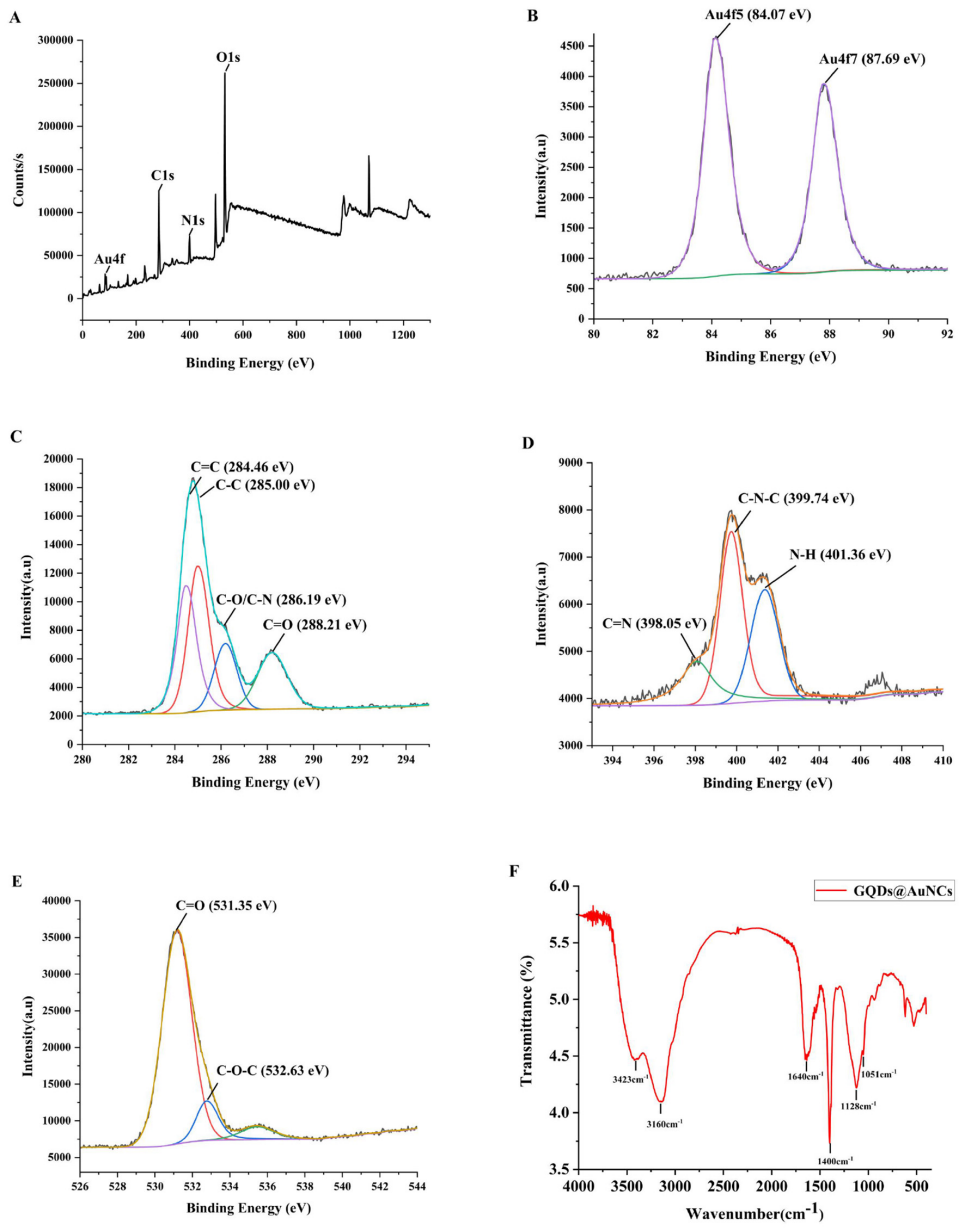
The full survey XPS spectra of GQDs@AuNCs **(A)**, Au4f pattern of GQDs@AuNCs **(B)**, C1s pattern of GQDs@AuNCs **(C)**, N1s pattern of GQDs@AuNCs **(D)**, O1s pattern of GQDs@AuNCs**(E)**, and FT-IR spectra of GQDs@AuNCs **(F)**.

The elemental composition of GQDs@AuNCs nanocomposites is shown in Figure 2, the signal peaks of Au4f, C1s, N1s, and O1s appear at 84, 285, 400, and 532 eV, respectively. There are two peaks at the Au4f signal, belonging to Au4f5 (84.07 eV) and Au4f7 (87.69 eV) (28). There are four peaks at the C1s signal, belonging to C=C (284.46 eV), C–C (285.00 eV), C–O/C–N (286.19 eV), and C=O (288.21 eV) (29). There are three peaks at the N1 s signal, belonging to C=N (398.05 eV), C–N–C (399.74 eV), and N–H (401.36 eV) (30). The peaks located at 531.35 eV and 532.63 eV at the O1s signal, belonging to C=O and C–O–C (31).

The surface functional groups of GQDs@AuNCs nanocomposites were shown in Figure 2F. The stretching vibration peaks of O–H, N–H, C=O and C–N bonds were observed near 3,423 cm^−1^, 3,160 cm^−1^ 1,640 cm^−1^ and 1,400 cm^−1^, respectively (31, 32); the stretching vibration peaks of C-O and S-Au were observed at 1128 cm^−1^ and 1,051 cm^−1^ (25, 33). To sum up, the results of FT-IR and XPS were in agreement.

Supplementary Figure S3A displays the UV absorption spectra of N-GQDs, G-AuNCs, and GQDs@AuNCs. N-GQDs and G-AuNCs exhibit strong absorption peaks at 299 nm and 400 nm, respectively. The GQDs@AuNCs nanocomposite also exhibits absorption peaks at the corresponding positions, with no new absorption peaks appearing, indicating that N-GQDs andG-AuNCs did not form new complexes during the self-assembly process. Supplementary Figure S3B displays the fluorescence spectrum scanning results of N-GQDs,G-AuNCs, and GQDs@AuNCs. The emission peaks of N-GQDs and G-AuNCs are located at 412 nm and 528 nm, respectively, at an excitation wavelength of 320 nm, while the GQDs@AuNCs nanocomposite exhibits emission peaks at both 412 nm and 528 nm. Compared with N-GQDs and G-AuNCs, the emission peak intensity of the GQDs@AuNCs nanocomposite at 412 nm is significantly enhanced, while that at 528 nm is weakened. This result indicates that fluorescence resonance energy transfer (FRET) occurs after the formation of the complex between N-GQDs and G-AuNCs (34).

### 3.2 GQDs@AuNCs ratiometric fluorescent sensor for detecting Al^3+^ and Cu^2+^

#### 3.2.1 Detection of Al^3+^ by the GQDs@AuNCs

**Figure 3:**
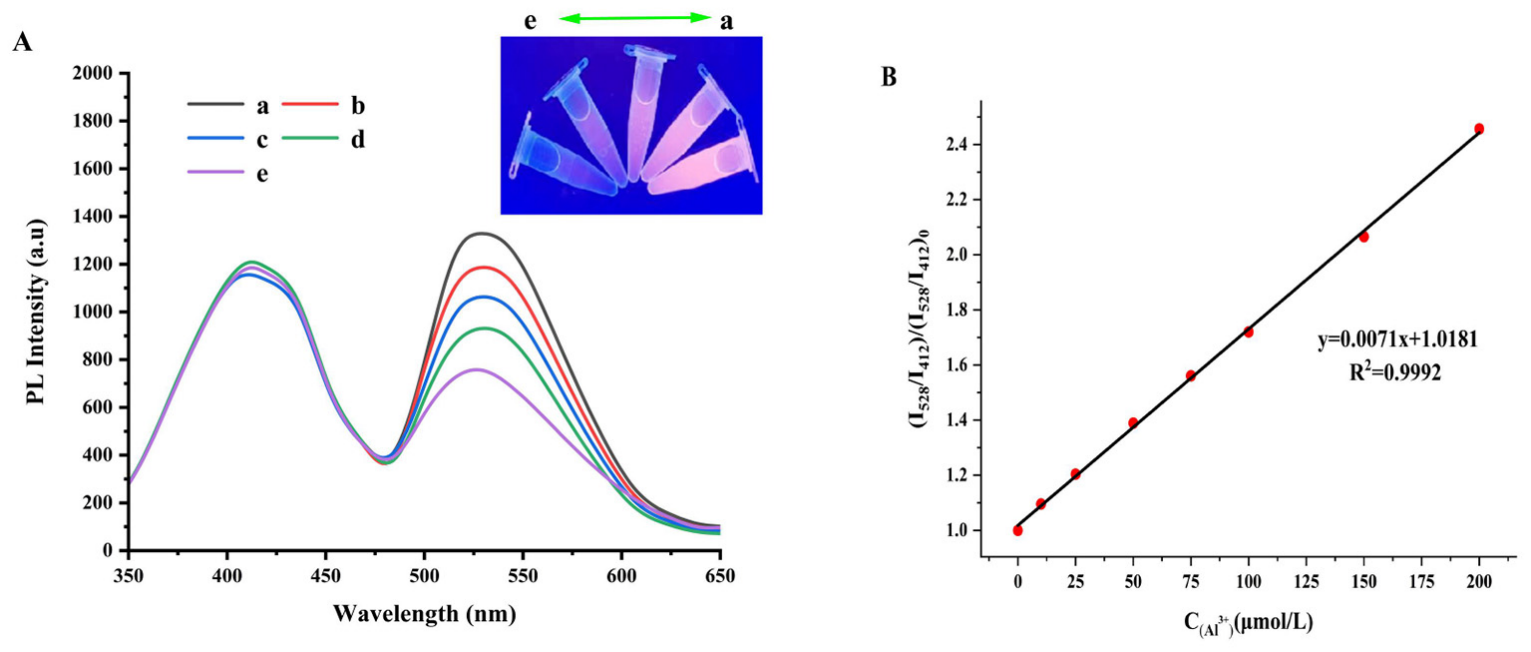
The fluorescence spectrum of the GQDs@AuNCs ratiometric fluorescent sensor for detecting Al^3+^ **(A)** (a-e represent Al^3+^ concentrations of 100,75, 50, 25, and 0 μM, respectively), and the standard curve of fluorescence intensity ratio (I_528_/I_412_)/(I_528_/I_412_)_0_ versus the concentration of Al^3+^ions **(B)**.

The fluorescence spectrum of the GQDs@AuNCs ratiometric fluorescent sensor for detecting Al^3+^ is shown in Figure 3A. The FI of GQDs@AuNCs gradually enhances as the concentration of Al^3+^ ions rises, and under UV light irradiation, the fluorescence color also undergoes a noticeable change from light purple to pink. When the concentration of Al^3+^ions is in the range of 1–200 μM, the FI ratio (I_528_/I_412_)/(I_528_/I_412_)_0_ shows a linear correlation with Al^3+^ concentration (*R*^2^ = 0.9992) (Figure 3B). The limit of detection (LOD) was 0.66 μM based on the equation LOD = 3σ/k, in which *k* is the slope of the standard curve and σ is the standard deviation of 11 fluorescence measurements. The phenomenon of significantly enhanced fluorescence of G-AuNCs induced by Al^3+^ may be attributed to the interaction between Al^3+^ and the surface ligands of G-AuNCs, which forms larger aggregated assemblies of G-AuNCs and thereby produces an aggregation-induced emission (AIE) enhancement effect (35). Furthermore, Al^3+^ binds to glutathione on the surface of G-AuNCs through coordinate bonds, resulting in the formation of G-AuNCs/Al^3+^ aggregates. This strong coordinate bonding can more effectively restrict the intramolecular vibration and rotation of G-AuNCs complexes, thereby generating a unique bond-induced emission (BIE) mechanism that enhances the fluorescence of G-AuNCs (36). In previous studies (Supplementary Table S1), Shao et al. (29) used a hydrothermal method to prepare bright green fluorescent NCDs capable of detecting Al^3+^, the FI of NCDs showed a linear correlation with Al^3+^ concentration (2.5–300 μM), with a LOD of 0.76 μM. Yasar et al. (37) created a new type of chemical probe (MPIM) with dual ratiometric fluorescent and colorimetric properties based on Schiff base derivatives, which can achieve the detection of Al^3+^. When MPIM interacts with different concentrations of Al^3+^, its LOD for Al^3+^ reach 12.6 μM and 1.82 μM. Luo et al. (35) synthesized glutathione (GSH)-capped Au NCs via a one-pot method. This fluorescence probe for Al^3+^ exhibited a wide detection range of 100–600 μM and excellent selectivity over other metal ions and common biomolecules. In this study, on the basis of synthesizing G-AuNCs, we further synthesized GQDs@AuNCs by utilizing the electrostatic interaction between G-AuNCs and N-GQDs. Compared with these studies (38–40), the GQDs@AuNCs synthesized in this study exhibit either a lower detection limit or a broader detection range.

#### 3.2.2 Detection of Cu^2+^ by the GQDs@AuNCs ratiometric fluorescent sensor

**Figure 4:**
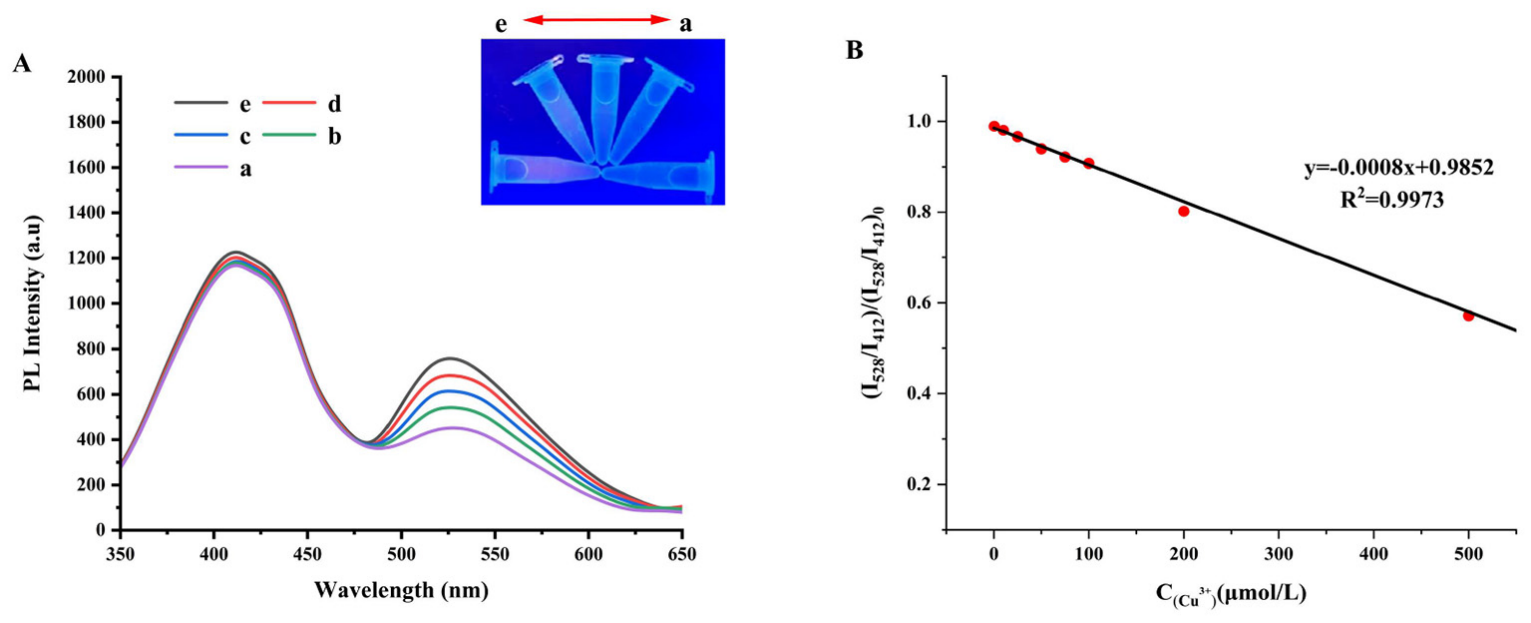
The fluorescence spectrum of the GQDs@AuNCs ratiometric fluorescent sensor for detecting Cu^2+^ **(A)** (a-e represent Cu^2+^ concentrations of 0, 25,50,75, and 100 μM, respectively), and the standard curve of fluorescence intensity ratio (I_528_/I_412_)/(I_528_/I_412_)_0_ versus the concentration of Cu^2+^ions **(B)**.

Figure 4A displays the fluorescence spectrum of Cu^2+^ detected by the GQDs@AuNCs ratiometric fluorescence sensor. The FI progressively rises with an increase in Cu^2+^ ion concentration. Moreover, under UV light irradiation, the fluorescence color also undergoes a visible change from purple to blue. The FI ratio (I_528_/I_412_)/(I_528_/I_412_)_0_ exhibits a strong linear correlation with Cu^2+^ concentration (*R*^2^ = 0.9973) when the concentration of Cu^2+^ ions is between 0.5 and 500 μM (Figure 4B). The LOD was evaluated to be 0.44 μM. Owing to ligand-to-metal electron transfer or ligand-to-metal-to-metal charge transfer, the triplet state of the metal center undergoes radiative transition, resulting in the fluorescence emission of AuNCs (41). The fluorescence quenching effect of Cu^2+^ on G-AuNCs may also rely on the charge transfer mechanism. Since glutathione on the surface (of G-AuNCs) contains abundant amino and carboxyl groups, there is a strong coordination interaction between these groups and Cu^2+^ (42). Upon the introduction of Cu^2+^ into the reaction system, GSH-Cu^2+^ complexes are formed; subsequently, charge transfers from G-AuNCs to Cu^2+^, leading to the enhancement of non-radiative transitions. This process directly results in a decrease in the fluorescence intensity of G-AuNCs until quenching occurs (43, 44). In previous studies (Supplementary Table S1) Jing et al. (45) utilized carbon dots (CDs) and hydroxyapatite (HAP) to assemble a nanocomposite fluorescent probe. The FI of this fluorescent probe exhibits a strong linear correlation with the Cu^2+^ concentration (10^−5^-10^−4^ M) and the LOD is 10 μM. Ramezani et al. (46) created a fluorescent probe grounded in 8-hydroxyquinoline chitosan silica precursor (HQCS), which can selectively detect Al^3+^ and Cu^2+^. For Cu^2+^, this probe has a detection range of 3.5–31 μM and the LOD is 1 μM. Li et al. (47) prepared a blue emission glutathione stabilized Au nanoclusters via ligand exchange with Au/histidine complexes, which were used for the detection of copper ions. The study found that glutathione-stabilized Au NCs exhibited a fluorescence emission intensity a hundred times higher than that of Au/histidine complexes and showed a highly selective fluorescence quenching response to copper ions. The fluorescence probe exhibits a detection range of 0.5–300 μM for Cu^2+^. In this study, on the basis of synthesizing G-AuNCs, we further synthesized GQDs@AuNCs by leveraging the electrostatic interaction between G-AuNCs and N-GQDs. Compared with individual G-AuNCs and the sensors reported in previous studies, the as-synthesized GQDs@AuNCs exhibits either a lower limit of detection (LOD) or a broader detection range (48, 49).

#### 3.2.3 Selectivity of GQDs@AuNCs for Al^3+^and Cu^2+^

Evaluating the selectivity of fluorescent sensors is a necessary condition for the successful construction of such sensors. As illustrated in Supplementary Figure S4, the FI ratio (I_528_/I_412_)/(I_528_/I_412_)_0_ in the presence of common representative metal ions (Fe^3+^, K^+^, Na^+^, Ca^2+^, Mn^2+^, Zn^2+^, Ba^2+^, and Mg^2+^) was analyzed to assess the selectivity toward Al^3+^ and Cu^2+^. The findings demonstrated that the FI of GQDs@AuNCs was not significantly changed by the introduction of metal ions other than Al^3+^ and Cu^2+^, suggesting that the fluorescent sensor exhibits high selectivity for Al^3+^ and Cu^2+^.

### 3.3 Detection of Al^3+^ and Cu^2+^ in real samples

The prepared GQDs@AuNCs ratiometric fluorescent sensor was evaluated for the detection of Al^3+^ in deep-fried dough sticks and fried dough twists, and Cu^2+^ in scallops and *Sinonovacula constricta* using the spiked recovery method. Certain concentrations (10 μM, 50 μM, 100 μM) of Al^3+^ and Cu^2+^ were added to the actual samples. Their fluorescence intensities were measured using a fluorescence spectrophotometer, and their contents were calculated. The recoveries of Al^3+^ in deep-fried dough sticks and fried dough twists are 99.44%−101.03% and 97.05%−100.48%, respectively, while the recoveries of Cu^2+^ in scallops and *Sinonovacula constricta* were 96.9%−102.96% and 90.04%−102.02%, respectively (Supplementary Table S2). The findings demonstrate that the GQDs@AuNCs fluorescence sensor can detect Al^3+^ and Cu^2+^ in food samples including deep-fried dough sticks, fried dough twists, scallops, and *Sinonovacula constricta* with excellent accuracy and precision.

## 4 Conclusion

On the whole, we have designed a novel ratiometric fluorescent sensor (GQDs@AuNCs) for detecting Al^3+^ and Cu^2+^, which is green, simple to synthesize, fast to react, and sensitive in detection. It detects Al^3+^ and Cu^2+^ with excellent stability and selectivity. With LODs of 0.66 μM and 0.44 μM, respectively, the FI ratio (I_528_/I_412_)/(I_528_/I_412_)_0_ exhibits an excellent linear relationship with the concentrations of Al^3+^ and Cu^2+^ ions within a specific concentration range. Additionally, it exhibits good anti-interference properties. The recoveries of Al^3+^ in deep-fried dough sticks and fried dough twists were 99.44%−101.03% and 97.05%−100.48%, respectively, while the recoveries of Cu^2+^ in scallops and *Sinonovacula constricta* were 96.9%−102.96% and 90.04%−102.02%, respectively. The GQDs@AuNCs ratiometric fluorescent sensor can quickly and sensitively detect Al^3+^ and Cu^2+^ in food, and thus has good application prospects.

## Data Availability

The raw data supporting the conclusions of this article will be made available by the authors, without undue reservation.

## Supplementary material

Supplementary Figure S1 The synthesis process and fluorescence staining change of GQDs@AuNCs.

Supplementary Figure S2 The Zeta potentials of N-GQDs, G-AuNCs, and GQDs@AuNCs.

Supplementary Figure S3 The UV absorption spectra of N-GQDs, G-AuNCs, and GQDs@AuNCs **(A)**, the fluorescence spectrum scanning of N-GQDs, G-AuNCs, and GQDs@AuNCs **(B)**.

Supplementary Figure S4 The fluorescence intensity ratio (I_528_/I_412_)/(I_528_/I_412_)_0_ of different metal cations (Al^3+^, Cu^2+^, Fe^3+^, K^+^, Na^+^, Ca^2+^, Mn^2+^, Zn^2+^, Ba^2+^, Mg^2+^).

Supplementary Table S1 Comparison of detection effects of different fluorescent sensors on Al^3+^ and Cu^2+^

Supplementary Table S2 Detection of Al^3+^ and Cu^2+^in real samples
