# Supplementary figures and images for: A ratiometric fluorescent sensor for Al3+ and Cu2+ detection in food samples
Source: Front Nutr. 2025 Nov 24;12:1707179. doi: 10.3389/fnut.2025.1707179 (PMC12682671; doi:10.3389/fnut.2025.1707179)

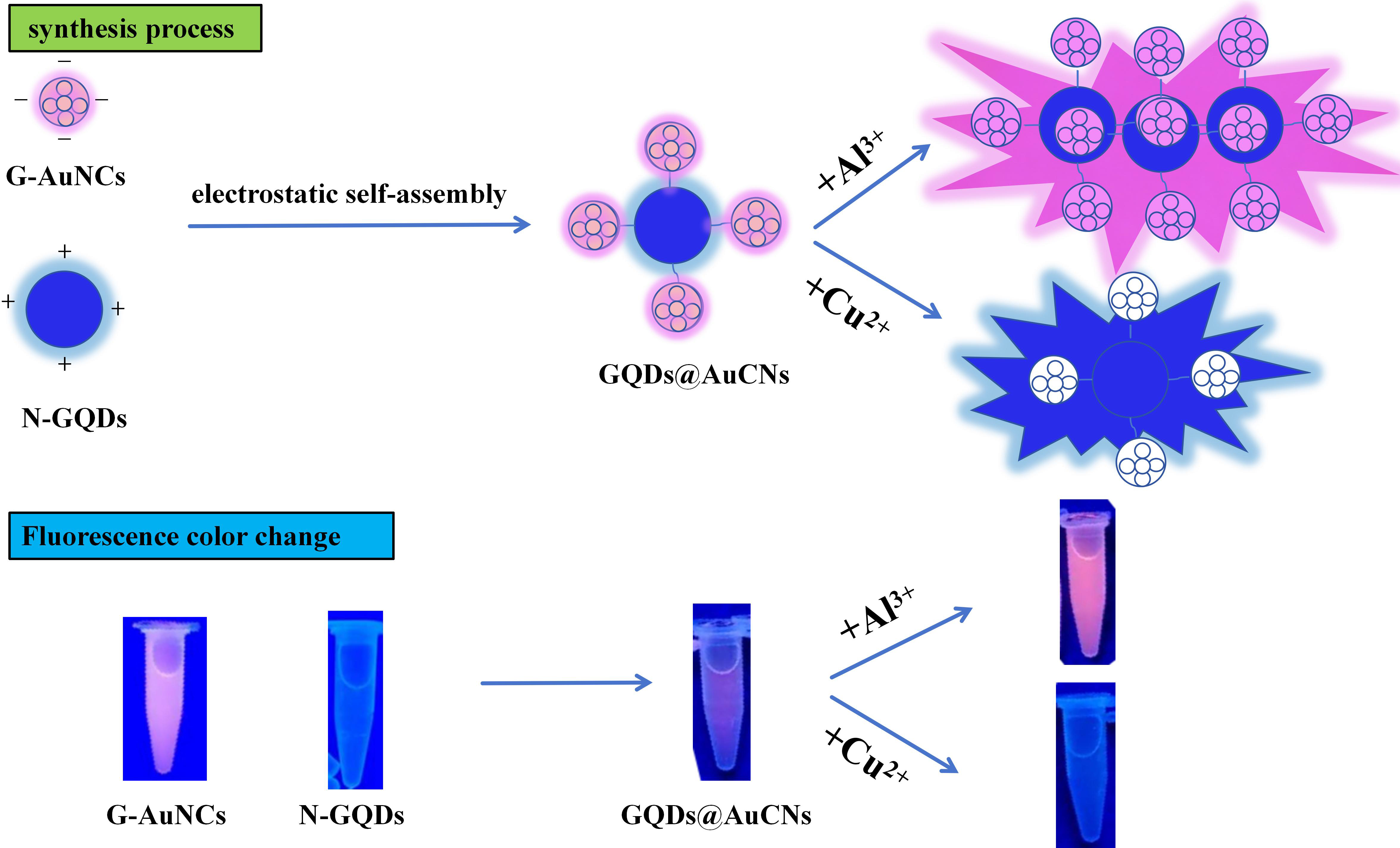

Supplement: Supplementary Figure S1 — The synthesis process and fluorescence staining change of GQDs@AuNCs. [file Image_1.jpeg]

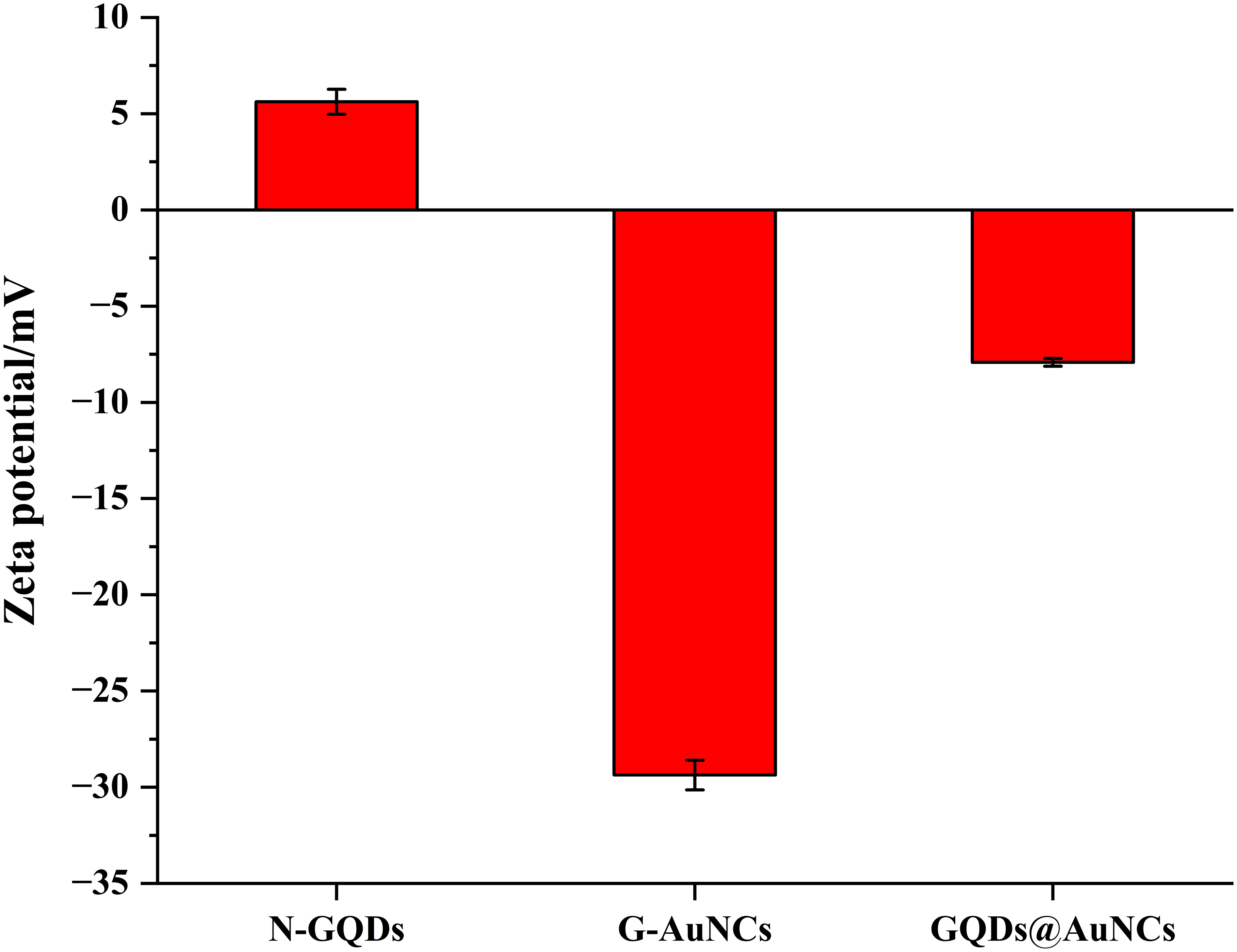

Supplement: Supplementary Figure S2 — The Zeta potentials of N-GQDs, G-AuNCs, and GQDs@AuNCs. [file Image_2.jpeg]

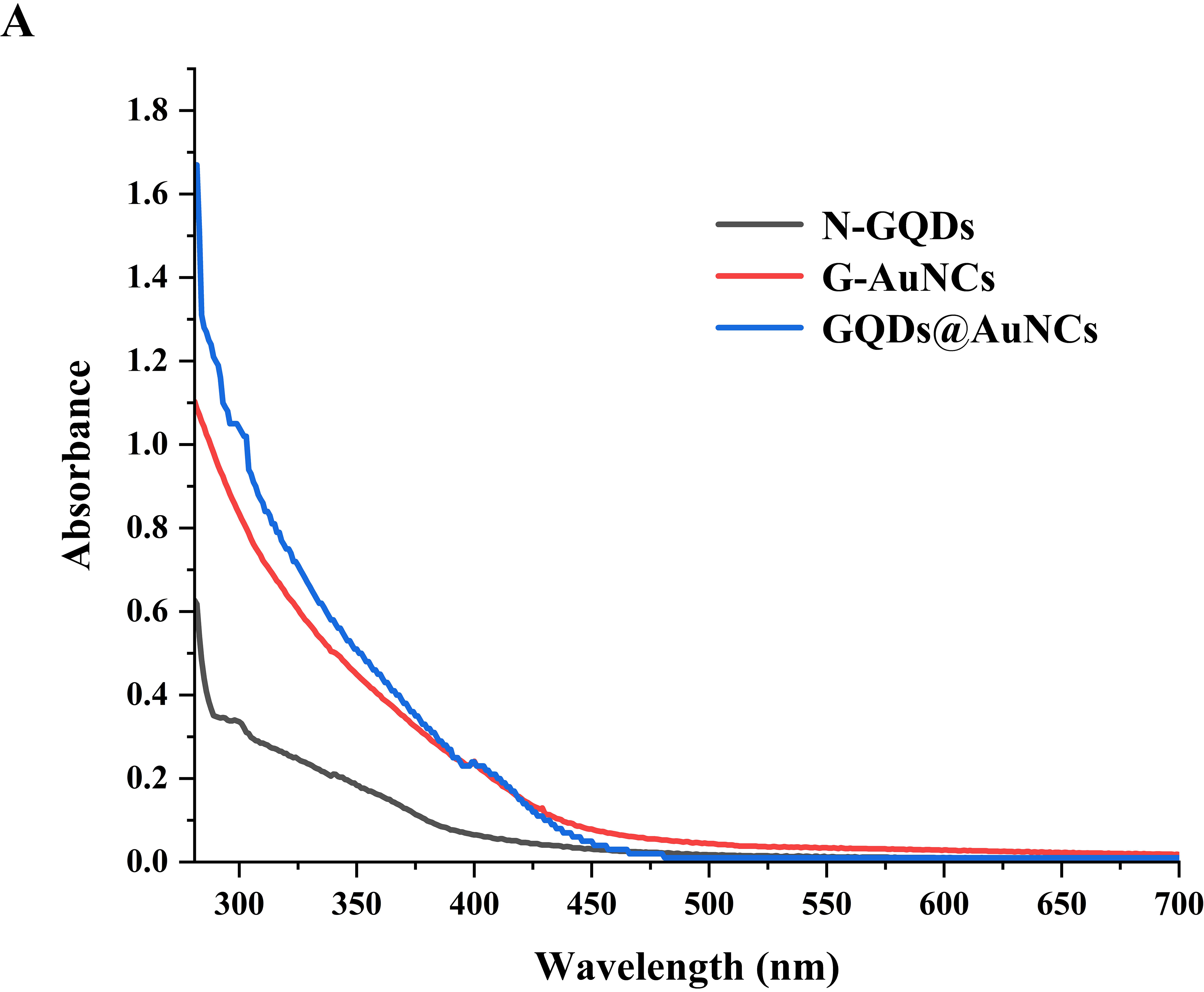

Supplement: Supplementary Figure S3 — The UV absorption spectra of N-GQDs, G-AuNCs, and GQDs@AuNCs (A), the fluorescence spectrum scanning of N-GQDs, G-AuNCs, and GQDs@AuNCs (B). [file Image_3.jpeg]

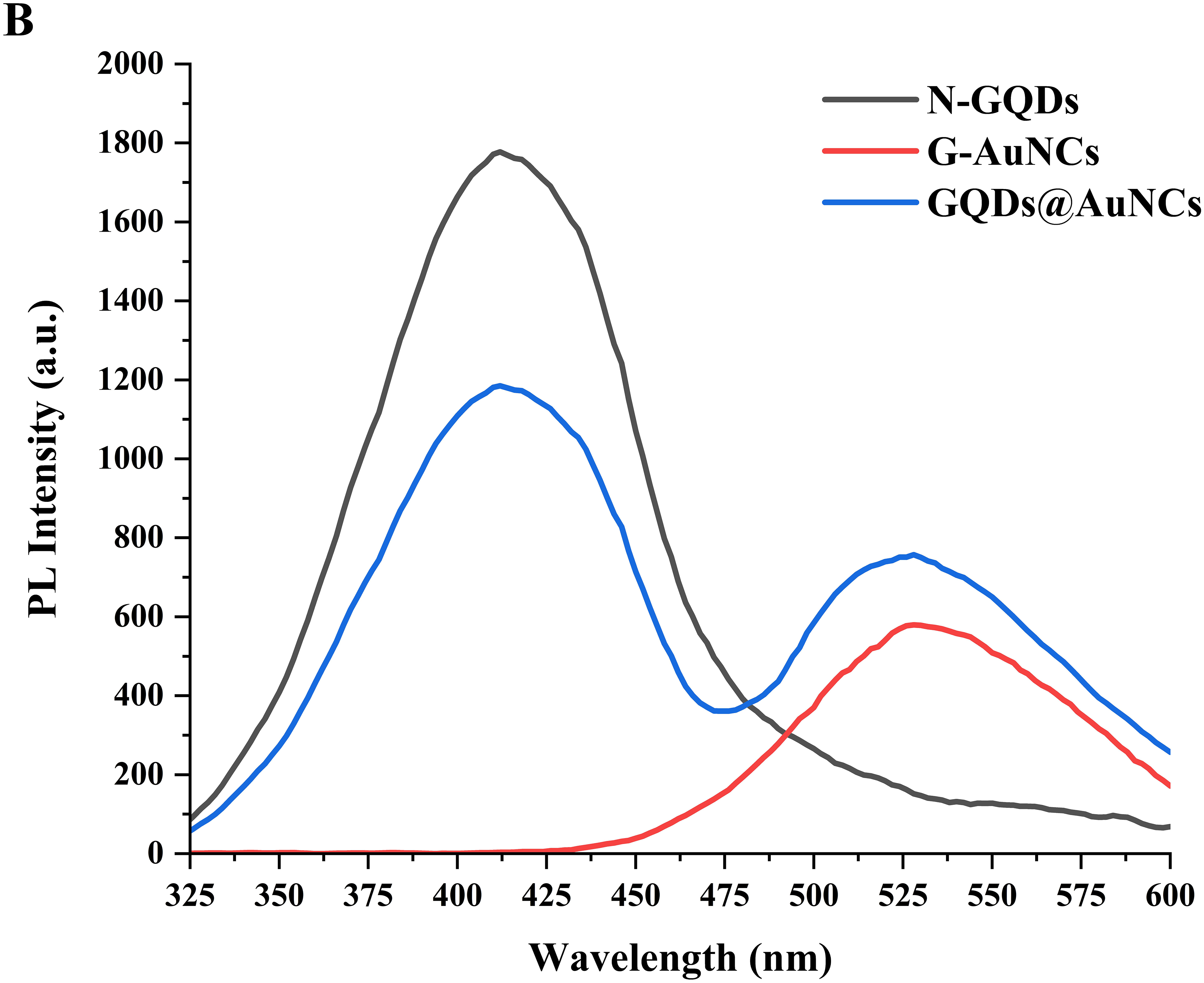

Supplement: Supplementary Figure S4 — The fluorescence intensity ratio (I528/I412)/(I528/I412)0 of different metal cations (Al3+, Cu2+, Fe3+, K+, Na+, Ca2+, Mn2+, Zn2+, Ba2+, Mg2+). [file Image_4.jpeg]

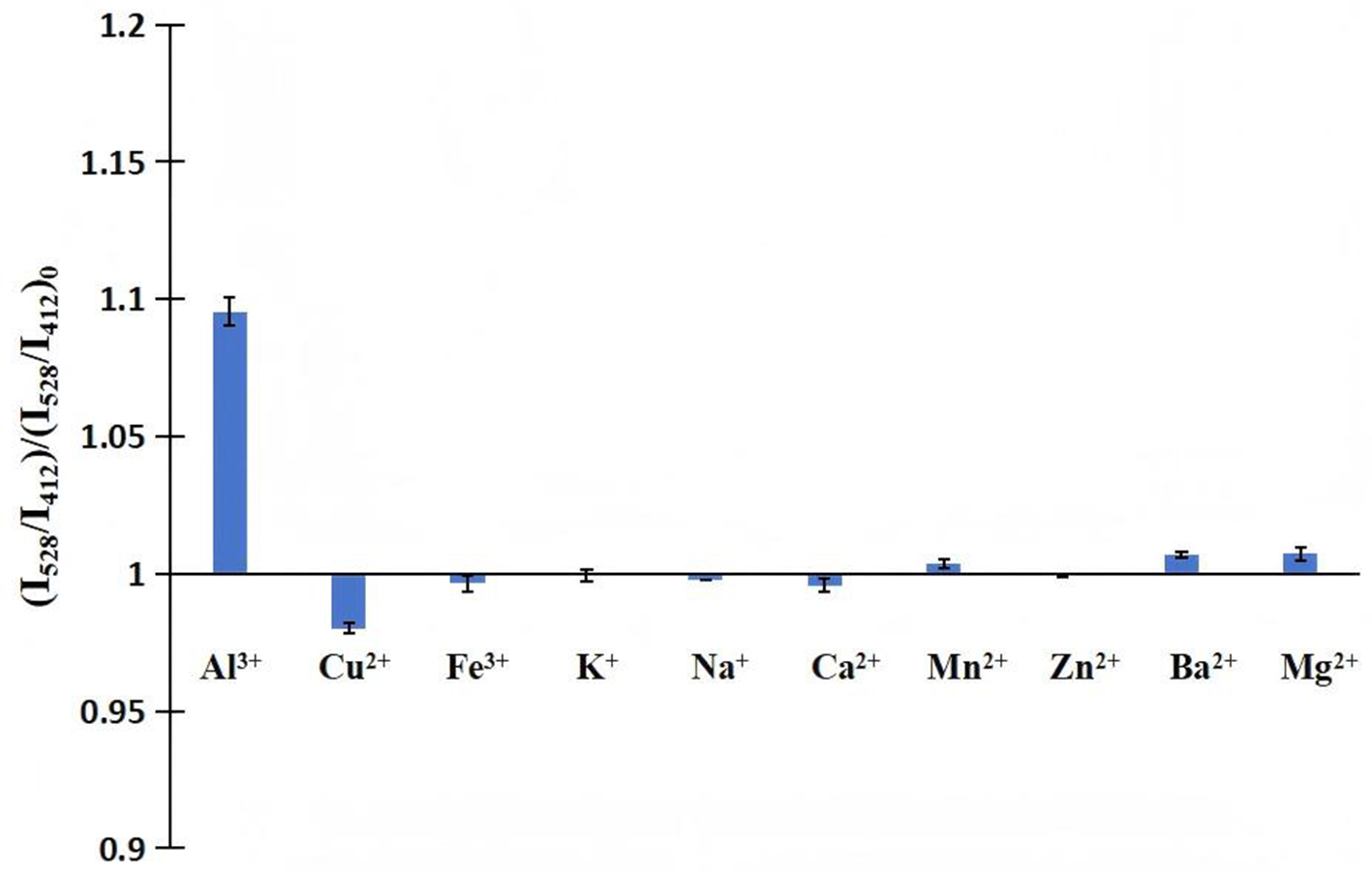

Supplement: Supplementary file 6 [file Image_5.jpeg]
