# Supplementary material for: A ratiometric fluorescent sensor for Al3+ and Cu2+ detection in food samples
Source: Front Nutr. 2025 Nov 24;12:1707179. doi: 10.3389/fnut.2025.1707179 (PMC12682671; doi:10.3389/fnut.2025.1707179)
Supplement: Supplementary Table S1 — Comparison of detection effects of different fluorescent sensors on Al3+ and Cu2+ [file Table_1.doc]

**Supplementary tables**

**Table S1.** Comparison of detection effects of different fluorescent sensors on Al³⁺ and Cu²⁺

**Table S2**. Detection of Al^3+^ and Cu^2+^in real samples

**Table S1.** Comparison of detection effects of different fluorescent sensors on Al³⁺ and Cu²⁺

| Sensors | Metal ion | Linear range  (μM) | Detection limit  (μM) | Ref. |
| --- | --- | --- | --- | --- |
| NCDs | Al^3+^ | 2.5–300 | 0.76 | [29] |
| MPIM | Al^3+^ | 25-150 | 12.6 and 1.82 | [30] |
| p(3-SH/DBPA/VPA/PEG-DA) | Al^3+^ | 7.43×10^-3^–0.11 | 6.3×10^-4^ | [38] |
| PFAP | Al^3+^ | - | 1.5 | [39] |
| NSDC-dots | Al^3+^ | 0-1.6 | 0.06 | [40] |
| GQDs@AuNCs | Al^3+^ | 1-200 | 0.66 | this work |
| GSH-AuNCs | Cu^2+^ | 0.5-300 | - | [47] |
| CH_3_CN: HEPES (3:2, v/v) | Cu^2+^ | 4-12 | 1.8 | [48] |
| probe (L) | Cu^2+^ | 0-40 | 0.0269 | [49] |
| CDs/HAP | Cu^2+^ | 10-100 | 10 | [36] |
| HQCS | Cu^2+^ | 3.5–31 | 1 | [37] |
| GQDs@AuNCs | Cu^2+^ | 0.5-500 | 0.44 | this work |

**Table S2**. Detection of Al^3+^ and Cu^2+^in real samples

| Sample | Metal ion | Addition amount  (μmol/L) | Detection amount  (μmol/L) | Recovery rate(%) |
| --- | --- | --- | --- | --- |
| Deep-fried dough sticks | Al^3+^ | 10 | 10.09±0.22 | 100.86% |
|  |  | 50 | 49.70±1.03 | 99.44% |
|  |  | 100 | 101.07±1.99 | 101.03 |
| Fried dough twists | Al^3+^ | 10 | 9.71±0.05 | 97.05% |
|  |  | 50 | 48.63±1.10 | 97.26% |
|  |  | 100 | 100.48±2.69 | 100.48% |
| Scallops | Cu^2+^ | 10 | 9.80±0.93 | 98.73% |
|  |  | 50 | 51.47±1.04 | 102.96% |
|  |  | 100 | 96.99±3.91 | 96.99% |
| *Sinonovacula constricta* | Cu^2+^ | 10 | 9.01±0.84 | 90.04% |
|  |  | 50 | 47.95±2.55 | 95.91% |
|  |  | 100 | 102.06±1.30 | 102.02% |
